# Transcriptomic and GC-MS Metabolomic Analyses Reveal the Sink Strength Changes during Petunia Anther Development

**DOI:** 10.3390/ijms19040955

**Published:** 2018-03-23

**Authors:** Yuanzheng Yue, Shaoze Tian, Yu Wang, Hui Ma, Siyu Liu, Yuqiao Wang, Huirong Hu

**Affiliations:** 1 Key Laboratory of Urban Agriculture in Central China, Ministry of Agriculture, Key Laboratory of Horticultural Plant Biology, Ministry of Education, College of Horticulture and Forestry Sciences, Huazhong Agricultural University, Wuhan 430070, China; yueyuanzheng@njfu.edu.cn (Y.Y.); tianshaoze@hotmail.com (S.T.); wangyuwuhan001@outlook.com (Y.W.); mahuiwuhan@hotmail.com (H.M.); liusiyuwuhan@hotmail.com (S.L.); wangyuqiaowuhan@hotmail.com (Y.W.); 2 Key Laboratory of Landscape Architecture, Jiangsu Province, College of Landscape Architecture, Nanjing Forestry University, Nanjing 210037, China

**Keywords:** *Petunia hybrida*, anther development, transcriptome, metabolome

## Abstract

*Petunia*, which has been prevalently cultivated in landscaping, is a dicotyledonous herbaceous flower of high ornamental value. Annually, there is a massive worldwide market demand for petunia seeds. The normal development of anther is the necessary prerequisite for the plants to generate seeds. However, the knowledge of petunia anther development processes is still limited. To better understand the mechanisms of petunia anther development, the transcriptomes and metabolomes of petunia anthers at three typical development stages were constructed and then used to detect the gene expression patterns and primary metabolite profiles during the anther development processes. Results suggested that there were many differentially-expressed genes (DEGs) that mainly participated in photosynthesis and starch and sucrose metabolism when DEGs were compared between the different development stages of anthers. In this study, fructose and glucose, which were involved in starch and sucrose metabolism, were taken as the most important metabolites by partial least-squares discriminate analysis (PLS-DA). Additionally, the qRT-PCR analysis of the photosynthetic-related genes all showed decreased expression trends along with the anther development. These pieces of evidence indicated that the activities of energy and carbohydrate metabolic pathways were gradually reduced during all the development stages of anther, which affects the sink strength. Overall, this work provides a novel and comprehensive understanding of the metabolic processes in petunia anthers.

## 1. Introduction

Anther is the key component of plant reproductive organs, for which any abnormal development processes will definitely influence the male plant fertility, and many studies have demonstrated that there are plenty of genes participating in anther development [1]. *Petunia* is a typical representative of the Solanaceae, which are located on a crucial node in plant phylogeny and have excellent ornamental characteristics and economic value in horticulture. Now, it has been widely used in plant molecular biology studies, as well as in gardens [2,3]. Different from the *Arabidopsis thaliana*, which has been taken as a model plant in fertility-related research, petunia has larger anthers and could be easily used in biochemical and histological analysis [4]. Hence, it has great potential to be taken as an ideal model species of dicots in studying anther development.

Recently, the functions of many key genes, including a series of transcription factors, participating in the anther/pollen development have been identified in model and crop plants [5]. However, the gene networks of anther development are very complex, and the understanding of their regulation still needs to be broadened [6]. Transcriptomic sequencing technology could be used to discover a great number of genes’ information, which has been widely utilized in anther transcriptome analysis of many species, such as in rice [7], wheat [8], maize [9], lily [10] and tobacco [11]. In petunia, the microarray and cDNA-amplified fragment length polymorphism (cDNA-AFLP) have shown that many anther/pollen-specific/predominant genes are expressed in the different development stages of anthers [12,13,14]. However, due to the limitations of these technologies, the transcriptomic data during petunia anther development are still fragmentary and call for more studies.

The gas chromatography-mass spectrometer (GC-MS) technique has been widely employed in metabolic profiling studies of plant tuber growth [15], fruit maturation [16], response to environmental changes [17] and disease [18,19]. The metabolomics of petunia floral scent production have also been well analyzed by this technique, in which benzaldehyde, phenylacetaldehyde, methyl benzoate, phenylethyl alcohol, iso-eugenol and benzyl benzoate were identified as the most abundant benzenoid compounds with the circadian emission rhythms [20]. Recently, two lines of *Petunia axillaris* (a strongly-scented line and a weakly-scented line) were used to profile the concentrations of metabolites and explore the regulation mechanism of the nocturnal emission rhythm of floral scent concentrations in the scent biosynthetic pathway [21]. However, this technique has not been reported to be used in the anthers, and the metabolic profiling of petunia anther development is still unclear. 

Omics based on high-throughput technologies have been a useful tool in exploring the complex profile of gene regulatory networks and analyzing the physiological and biochemical changes of metabolic pathways. Recently, many biological phenotypes have been deeply analyzed using the transcriptomic and metabolomic integration strategy, such as the accelerated fruit senescence mechanism after cold storage in litchi [22], the adaptive responses to ultraviolet radiation of grapevine [23], the sugar and organic acid metabolism and puffing disorder of *Citrus* fruit [24], the copper stress acclimation of brown algae (*Ectocarpus siliculosus*) [25] and the transcriptional regulation of the metabolism of sulfate starvation and resupply in *Arabidopsis* [26].

In order to broaden the knowledge of gene regulation networks and the metabolite profiling during petunia anther development, herein, the transcriptomic and metabolomic of petunia anthers at three critical development stages were analyzed using RNA-seq and GC-MS technologies, in which many significantly differentially-expressed unigenes (DEGs) and metabolic pathways, as well as critical metabolites were identified. This is the first time that comparative multi-omics systems have been used to explore the development process of petunia anthers, and we hope it will be helpful in the following anther development research.

## 2. Results

### 2.1. The Chlorophyll Content Is Reduced during the Anther Maturation Process 

**Figure 1:**
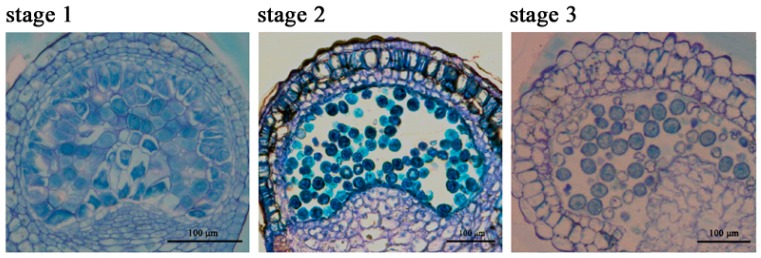
Semi-thin sections of petunia “Fantasy Red” anthers. Stage 1, Stage 2 and Stage 3 mean pollen mother cell, microsporogenesis and pollen grain stages, respectively, for which the anthers are from flower buds with a bud length (without sepal) of 3 ± 0.5 mm, 15 ± 0.5 mm and 35 ± 0.5 mm individually. Scale bars represent 100 μm.

**Figure 2:**
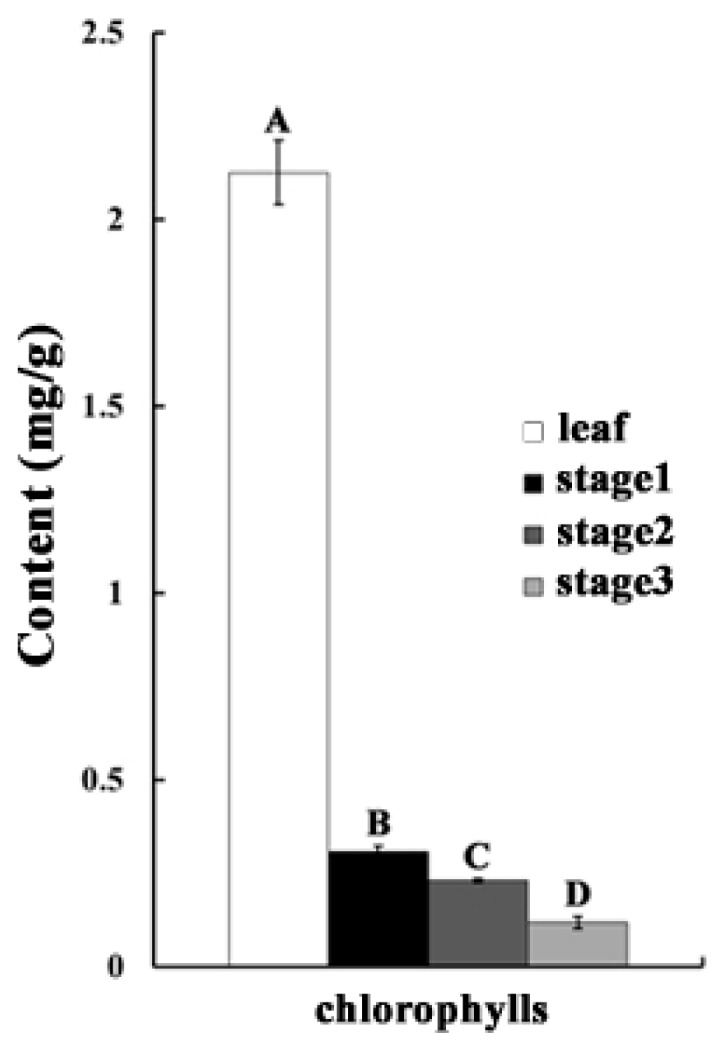
Contents of chlorophyll in petunia anthers and leaves. Stage 1 means the anthers from the 3 ± 0.5 mm flower buds; Stage 2 means the anthers from the 15 ± 0.5 mm flower buds; Stage 3 means the anthers from the 35 ± 0.5 mm flower buds. Different capital letters in each column stand for a significant difference at 0.01 level.

Bulleted in this study, the chlorophyll content was successfully tested in pollen mother cell stage (Stage 1), microsporogenesis stage (Stage 2) and pollen grains stage (Stage 3) of petunia anthers (Figure 1). The content of chlorophyll has a significant decreasing tendency with the development of anthers (Figure 2). When compared with leaf, the concentration of chlorophyll is much less, though it has been proven that chlorophyll indeed exists in anthers, and chlorophyll could play important roles in anther development.

### 2.2. Transcriptome Sequencing

**Figure 3:**
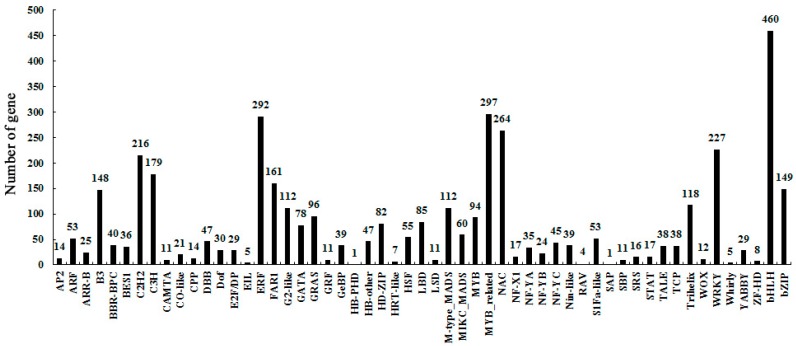
Identified transcription factors (TFs) during petunia anther development.

Three total RNA samples were isolated from petunia anthers of different development stages (Stage 1, Stage 2, Stage 3). These RNA samples were at a concentration of about 2000 ng·μL^−1^ with OD_260/280_ ≥ 1.8, and the RNA integrity numbers (RINs) of 9.1–9.6 were used for cDNA libraries’ construction. The three cDNA libraries were sequenced using the Illumina HiSeq^TM^ 2000 platform. A total of about 162 million raw sequencing reads was generated, and after discarding the low-quality reads, we obtained about 158 million clean reads, 97.53% of the raw reads. For all three samples, the quality score above 20 (Q20) was around 98.50%, and the GC percentages were 43.48%, 43.26%, 43.46%, respectively. We achieved 89.7%, 90.2% and 89.8% genome alignment ratios of unigenes in Stage 1, Stage 2 and Stage 3 of petunia anthers, respectively (Table S1). Approximately 82% (27,152) of the genes were detected to be expressed in the three stages of anther development compared to the total predicted genes (32,928) of the petunia genome. At last, a total of 4118 putative transcription factors (TFs), which belonged to 55 TF families, were identified from the database of transcriptomes (Figure 3).

### 2.3. Identify Differentially-Expressed Unigenes between Anther Transcriptomes

**Figure 4:**
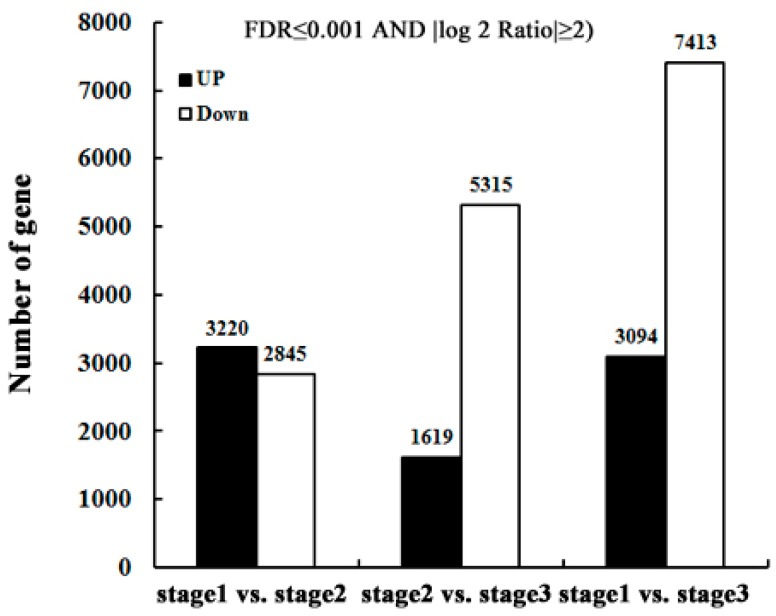
Statistics of differentially-expressed genes (DEGs) showing up- (black) or down- (white) regulation between the samples.

The DEGs in pairs of two stages of anther maturation progress are shown in Figure 4. The transcriptome of the pollen grain Stage 2 in comparison to the microsporogenesis Stage 1 is characterized by 6065 DEGs, of which 3220 are upregulated unigenes and 2845 downregulated. As the anthers develop from the microspores of Stage 2 to the mature pollen of Stage 3, 6934 unigenes are DEGs, of which 1619 are upregulated and 5315 downregulated. Remarkably, when compared with Stage 1, Stage 3 had 3094 upregulated unigenes and 7413 downregulated unigenes. The comparison between Stage 1 and the other anther developmental stages (2 and 3) implied that the number of downregulated unigenes significantly increased with the pollen formation.

### 2.4. KEGG Pathways Enrichment

According to KEGG annotation, the DEGs among the anther transcriptomes were mainly assigned to 127 pathways. The upregulated and downregulated genes were used in the KEGG pathway enrichment analysis, respectively. Based on the KEGG pathway enrichment analysis of the upregulated expressing genes, the “photosynthesis” and “starch and sucrose metabolism” pathways were significantly different as compared within each of two transcriptomes (Stage 1 with Stage 2, Stage 1 with Stage 3, Stage 2 with Stage 3). Interestingly, the KEGG pathway enrichment analysis of the downregulated genes between each of two transcriptomes also obtained the same results. The statistically-enriched pathways between each two transcriptome are shown in Figures S1–S3.

### 2.5. Validation of the Gene Expression Profiles by qRT-PCR

**Figure 5:**
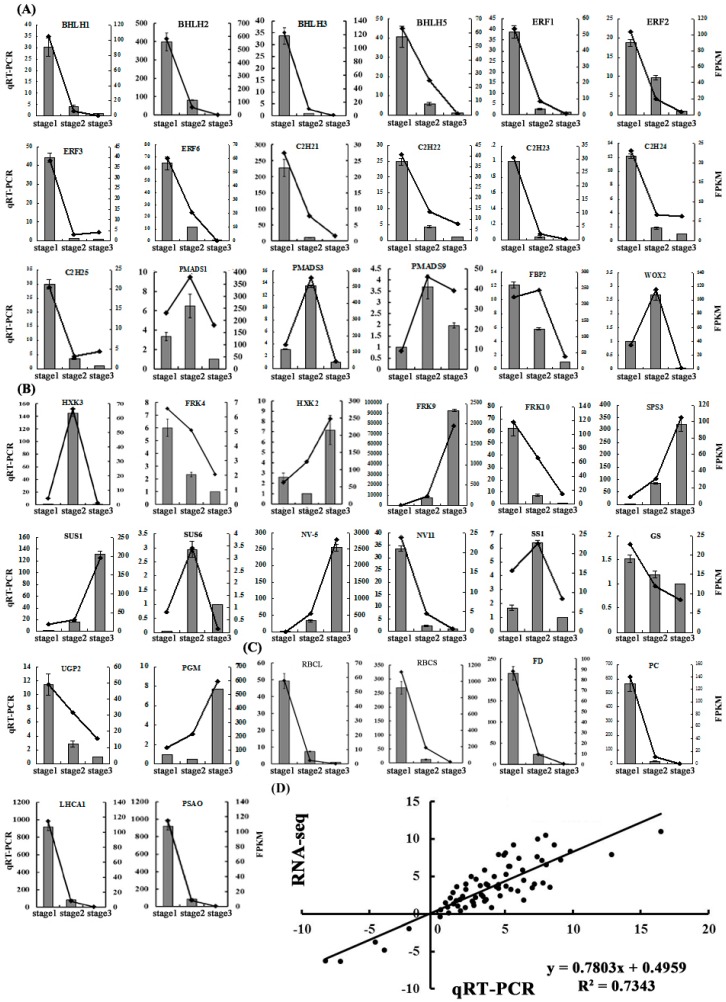
The qRT-PCR validation of DEGs. The relative expression levels of 18 transcription factors (**A**), 14 starch and sucrose metabolism pathways (**B**) and 6 photosynthesis related genes (**C**). The left *Y* axis represents the relative transcript amount obtained by qRT-PCR. The right *Y* axis represents the fragments per kb per million fragments (FPKM) value of each gene using RNA-Seq analysis. Error bars indicate the standard errors. (**D**) Correlation analysis of the gene expression ratios between qRT-PCR and RNA-seq.

In order to confirm the transcription profile revealed by RNA-seq data, 18 transcription factors, 14 starch and sucrose metabolism pathways and 6 photosynthesis-related genes were selected to design specific primers for qRT-PCR analysis (Figure 5A–C). The overall correlation coefficient (*R* = 0.89) between RNA-seq and qRT-PCR data was obtained by linear regression analysis, which showed a good correlation between these two data (Figure 5D), indicating that the three transcriptomics data were reliable.

### 2.6. The Expression Patterns of Photosynthesis-Related Genes

**Figure 6:**
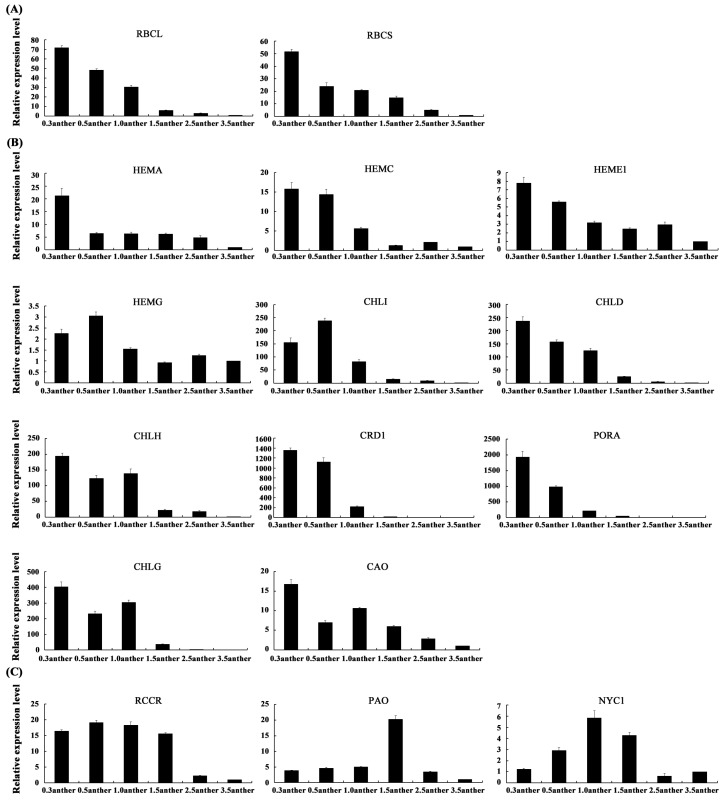
Expression analyses of the photosynthesis-related genes during petunia anther development. The expression patterns of 2 photosynthesis ratio genes (**A**), 11 chlorophyll biosynthesis genes (**B**) and 3 chlorophyll degradation genes (**C**) in all the petunia anther development stages. The sample of 0.3anther is at the pollen mother cell stage; the samples of 0.5anther, 1.0anther, 1.5anther are at the microspore stage; the samples of 2.5anther and 3.5anther are at the pollen grain stage.

In this study, two key enzyme genes *RBCL* and *RBCS*, which play important roles in controlling the photosynthesis rate, were used to explore their expression profiles during the different anther development processes. The results showed that their expression levels were very high in the initial development stage of anthers and then decreased gradually with the maturing of anthers (Figure 6A). Then, 11 enzyme genes related to chlorophyll biosynthesis were selected to test their expression patterns. Interestingly, they had an overall downregulated expression trend, which was very similar to *RBCL* and *RBCS* (Figure 6A,B). Lastly, the results of the three chlorophyll degradation genes showed that, from the mother cell stage until the microsporogenesis stage, the expression levels of *RCCR* remained high, and *NYC1* increased gradually, while *PAO* remained low until a sudden increase occurred in the microsporogenesis stage. After that, expressions of all three genes significantly decreased in the 2.5-cm stage and remained low at the pollen grain stage (Figure 6C).

### 2.7. Metabolome Analysis of Petunia Anther Development by GC-MS 

**Figure 7:**
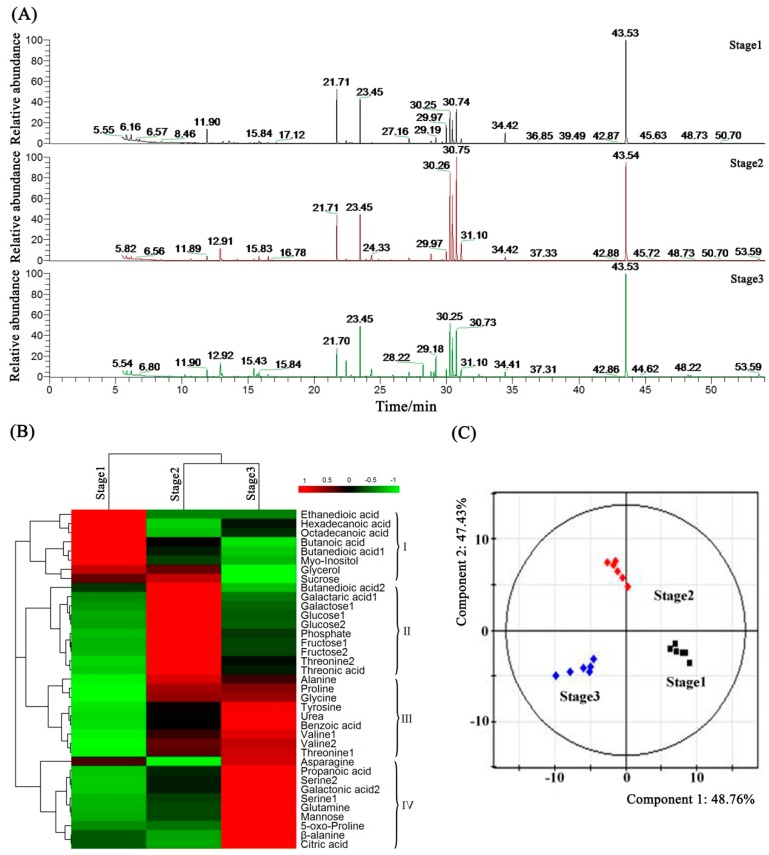
GC-MS metabolomic analysis of petunia anthers. (**A**) GC-MS total ion chromatography of anthers under Stage 1, Stage 2 and Stage 3; (**B**) clustering of the 37 identified metabolites analyzed by GC-MS; (**C**) the PLS-DA analysis of metabolites in different anther development stages of petunia.

To investigate the metabolic profile changes of anthers in the maturation processes, we obtained the GC-MS total ion current (TIC) chromatograms for 18 petunia anther samples from three typical development stages. The obvious differences of chromatographic peaks were observed between sample groups, and the retention times were fairly consistent and reproducible (Figure 7A). In this research, a total of 37 metabolites out of 126 total peaks could be identified in our sample libraries across all samples, and most were either amino acids or carbohydrates.

### 2.8. Metabolic Profiling and Core Differential Metabolites’ Identification

These identified metabolites were visualized in HeatMap, in which they were generally classified into four clusters according to their expression data (Figure 7B). Clusters I, II, III and IV featured the high enrichment of metabolites at anther Stage(s) 1/and 2, only 2, 2 and 3 and only 4, respectively. In Stage 1, the most abundant metabolites were organic acids (ethanedioic acid, hexadecanoic acid, octadecanoic acid, butanoic acid and butanedioic acid) and alcohols (myo-inositol and glycerol), and the abundance of amino acids and sugars (fructose, glucose and galactose) was very low. The greatest abundance of sugars (fructose, glucose, galactose and sucrose) was in Stage 2, then their contents were significantly reduced in Stage 3; while other organic acids and nearly all of the amino acids had the greatest enrichment in Stage 3 (Figure 7B).

To assess systematically the metabolic profile changes during anther development, the PLS-DA plot was generated from the GC-MS metabolite data of Stage 1, Stage 2 and Stage 3 anthers and showed clear metabolic differences between two stages. Remarkably, Stage 1, Stage 2 and Stage 3 anthers could be completely separated sufficiently by use of two principal components. The first principle component (PC1) accounting for 48.76% of the variation in the data could separate all three types of anthers with no outliers, and the second component (PC2) accounting for 47.43% of the variation could result in clear separation of Stage 2 anthers from the others (Figure 7C). The contribution of each variable to PC1 and PC2 was also calculated by giving each variable a weight value. The top 10 core differential metabolites of PC1 and PC2 discrimination are illustrated in Table S2. Interestingly, the two most important core metabolites were fructose (relative levels of 1.00, 5.66 and 2.20 in Stage 1, Stage 2 and Stage 3 samples, respectively) and glucose (relative levels of 1.00, 6.24 and 1.95 in Stage 1, Stage 2 and Stage 3 samples, respectively). Both of their contents were significantly increased from Stage 1 to Stage 2 and then obviously decreased in Stage 3 (Figure 7B).

## 3. Discussion

*Petunia hybrida*, which derived from crosses between two wild parents *P*. *axillaris* and *P*. *inflata*, is the most popular bedding plant in the world. Furthermore, it has also been taken as a model species for research on biological phenomena. Recently, the high-quality assembled reference genomes of these two wild parents have been accomplished, and this will directly accelerate further research into petunia’s unique characterization [27]. As one of the most important reproductive organs, the development of anther is a complex process, and any disturbing of it could significantly influence the pollen’s fertility, which would bring great losses in breeding. The previous results implied that petunia anther development is a complex process, and some anther-/pollen-specific genes/promoters were obtained [7,12,28,29]. However, due to the different concerns of experiment designs and technical limitation, the knowledge related to petunia anther development still needs to be refined.

In this study, three typical development stages of petunia anther, which are the pollen mother cell, microsporogenesis and mature pollen grains stages [30], were selected to sequence and identify gene expression at the whole genome level using Illumina sequencing technology. It has been known that the basic helix-loop-helix protein (bHLH) transcription factors that participate in various development processes constitute one of the largest families in plants [31]. Here, a total of 55 TF families were identified from the database of the transcriptomes, of which the largest absolute number of TFs (460) was from the members of the bHLH family (Figure 3). These data indicated that the bHLH TFs could have key functions in controlling petunia anther development. When comparing to the abundance of DEGs between two different anther development stages, the number of downregulated genes was significantly increased during anther maturation, and on the contrary, the number of upregulated genes had a decreasing trend (Figure 4), implying that the genes’ expression and the activity of anther in the initial development stage are more vigorous than the later stages. KEGG pathway enrichment analysis was employed to further analyze the biological functions of DEGs. The “photosynthesis” and “starch and sucrose metabolism” pathways relevant to energy metabolism were enriched in both groups (Figures S2–S4). This result suggested that these pathways could play critical roles in petunia anther development. In this research, the whole anthers, which contain a high percentage of biomass from non-pollen tissues, were taken as samples to implement the transcriptome analysis. Nowadays, laser microdissection technology has made it possible to exactly isolated target tissues of pollen. Without the once green anther cuticle, neither “photosynthesis”—nor “starch and sucrose metabolism”—related genes/pathways had been found important in pollen development [32,33].

There are many enzymes participating in chlorophyll biosynthesis, and any disruption of these would seriously restrain the synthesis of chlorophyll, which could significantly influence the photosynthesis rate [34]. In this research, the expression of 11 enzyme genes related to chlorophyll biosynthesis featured sustained declines (Figure 6B), implying that the ability of chlorophyll biosynthesis was gradually weakened during anther development. It has been elucidated that chlorophyll degradation is usually remarkably initiated at the final stage of a plant organ development process, such as fruit ripening and leaf senescence [35,36]. However, it has not been reported in plant reproductive organs, and the molecular mechanism of chlorophyll degradation remains unclear. Through transcriptomic analysis, a series of chlorophyll degradation-related genes was identified in petunia anthers. Dramatically, the expression patterns of three typical chlorophyll degradation pathway-related genes (*RCCR*, *NYC1* and *PAOs*) were not strictly negative correlated with the chlorophyll biosynthesis-related genes, and all of them were significantly reduced before the microsporogenesis stage (Figure 6C). These results implied that the molecular mechanism of chlorophyll degradation is fairly complex in petunia anthers, and further exploration of this metabolic pathway during petunia anther development is important. Interestingly, chlorophyll was also detected in anthers, and the content decreased with anther growth (Figure 2). The *RBCL* and *RBCS* genes, which encode ribulose 1,5-bisphosphate carboxylase/oxygenase (RuBisCO), could control the photosynthesis rate by CO_2_ fixing [37]. Here, the expression levels of these genes also showed a decreased trend during the maturing processes of anthers (Figure 6A). These results suggested that the photosynthesis pathway existed in petunia anthers and was significantly active in the early development stages.

It has been reported that the *INVERTASE* (*INV*) gene could catalyze the hydrolysis of sucrose to glucose and fructose, and the *HEXOKINASE* (*HK*) and *FRUCTOKINASE* (*FK*) genes participate in the glycolysis process of glucose and fructose [38,39]. Here, the expression patterns between *INV* with *HK* and *FK* were completely opposite (Figure S4). This result indicated that the production of glucose and fructose was most active at the beginning of petunia anther development, and then, the glycolysis ratios of glucose and fructose were significantly enhanced, causing the increasing consumptions of glucose and fructose in the following development stages. This conclusion also has been confirmed by the metabolomic analysis, in which the contents of glucose and fructose were firstly increased and then decreased with the development of anthers (Figure 7B).

The first component (PC1) and the second component (PC2) of the variation in the data could separate all three stages of anthers with no outliers (Figure 7C), indicating that our metabolomic analysis was reliable and could sufficiently reflect the metabolic profile changes of anthers. A previous study has proven that the sugar signaling pathway might be a central signaling network of cotton anthers, and the conversion of sucrose to fructose and glucose could be suppressed under high temperature condition [39]. Interestingly, the top two core different metabolites during the anther development processes were confirmed as fructose and glucose (Table S2), which were two critical materials in the starch and sucrose metabolism pathways, indicating that this pathway could be critically important in petunia anther development. This finding is in accordance with our above KEGG pathway enrichment result. These evidence suggested that the sink strength was altered in the different development stages of petunia anthers. It is worth mentioning that many peaks detected through GC-MS analysis are still unidentified, and these unknown metabolites would be the subject of our future studies.

## 4. Materials and Methods 

### 4.1. Plant Materials 

Plants of *Petunia hybrida* “Fantasy Red” are a multi- and small-flowered cultivar that was grown in a mix of peat moss, perlite and vermiculite at a volume ratio of 2-1-1 in the experimental greenhouse of Huazhong Agricultural University. Anthers were selected for the pending analysis at immature (pollen mother cell), intermediate (microsporogenesis) and mature (pollen grain) stages, which represent three typical stages of petunia anther development, measured as flower bud lengths (sepals were not included) of 3 ± 0.5 mm (Stage 1), 15 ± 0.5 mm (Stage 2) and 35 ± 0.5 mm (Stage 3), respectively (Figure 1). In order to satisfy the basic sample weight of every experiment, anthers of three plants were mixed with equal amounts and taken as one biological replicate. The fresh samples were taken and immediately frozen in liquid nitrogen and stored at −80 °C until analysis.

### 4.2. Chlorophylls and Carotenoids Content Assay

Briefly, 100 mg of mature leaf and different anther (Stage 1, Stage 2 and Stage 3) tissues of each replicate plant were extracted with 5 mL 96% (*v*/*v*) ethanol to determine the content of chlorophyll. Extraction was performed in the dark for 24 h at 4 °C and then analyzed by spectrophotometry using a Hitachi U-2000 spectrophotometer. The absorbance readings were performed at 665 nm and 649 nm for chlorophyll a and chlorophyll b, respectively. Contents of chlorophyll a (C_a_), chlorophyll b (C_b_) and chlorophylls (P_chlorophylls_) were calculated according to the following equation: C_a_ (mg/mL) = 1.95 × A_665_ − 6.88 × A_649_, C_b_ (mg/mL) = 24.96 × A_649_ − 7.32 × A_665_ and P_chlorophylls_(mg/g) = 50 × (C_a_ + C_b_), where A represents absorbance at the specified wavelength. Every experiment was repeated with three biological replicates.

### 4.3. RNA Extraction 

Total RNA was extracted from the anther samples above using RNAiso Reagent (Takara, Tokyo, Japan) according to the previously described method [40]. The quality of total RNA samples was assessed using 1% agarose gels. The concentration of RNA samples was tested using NanoDrop (Thermo Scientific, Waltham, MA, USA). The RIN value of RNA was determined by the Agilent 2100 Bioanalyzer (Agilent Technologies, Santa Clara, CA, USA).

### 4.4. RNA-Seq and Reads Mapping 

Three petunia anther cDNA libraries of three different developmental stages (Stage 1, Stage 2 and Stage 3) were prepared using the TruSeq RNA Sample Preparation Kit (Illumina, San Diego, CA, USA) following the manufacturer’s instructions. After the purification and fragmentation of RNA, cDNA synthesis, end repair, adapter ligation and PCR amplification, the cDNA library products (ranging from 200–700 bp) were sequenced on the Illumina HiSeq^TM^ 2000 instrument using paired-end sequencing technology by the staff at Beijing Genome Institute (BGI) (Shenzhen, China). The datasets involving three different anther stages were deposited in the NCBI Sequence Read Archive (SRA) with Accession Number SRP126902 under BioProject Number PRJNA422657. Before assembly, the raw reads were first filtered to obtain high-quality clean reads by removing adapter sequences, reads with more than 20% low-quality bases (quality value < 20) or ambiguous nucleotides (denoted with an “N” in the sequence trace). After the purifying process, the clean reads were mapped on the *Petunia axillaris* reference genome (https://solgenomics.net/organism/Petunia_axillaris/genome), which is more closely related to *P*. *hybrida* “Fantasy Red” than other sequenced species *Petunia inflata*. The SOAP2 software (http://soap.genomics .org.cn/soapaligner.html) was used to map the clean reads under strict default parameters (mismatch number ≤ 5). The reference genes mapped by at least one read, in at least one sample, were defined as expression genes and selected for further analysis. The Pfam database of the petunia anther transcriptome was obtained from the reference annotation of the *P*. *axillaris* genome.

### 4.5. Gene Expression Difference Analysis

The unigene expression level was calculated using the FPKM method (fragments per kb per million fragments) [41]. The significance of unigenes’ expression difference was determined by using “FDR ≤ 0.001 and the absolute value of log_2_Ratio ≥ 2” as the threshold [42,43]. All the upregulated or downregulated genes were mapped to the KEGG pathway database, and the numbers of unigenes for every KEGG Orthology (KO) term were calculated. Significantly enriched KO terms from the set of upregulated or downregulated genes were identified using the formula of the hypergeometric test [44], when comparing these upregulated or downregulated genes with the whole petunia anther transcriptome background.

### 4.6. qRT-PCR Analysis

A TransScript One-Step gDNA Removal and cDNA Synthesis SuperMix kit (Transgene, Shenzhen, China) was used to synthesize the first-strand cDNA on the basis of the manufacturer’s instructions. The qRT-PCR was carried out using the SYBR Premix Ex Taq™ IIkit (Takara, Tokyo, Japan) in the ABI 7500 Fast Real-Time PCR System (Applied Biosystems, Foster City, CA, USA) according to the protocol described previously [28]. The gene expression level was calculated by the 2^−ΔΔ*C*t^ method, with the *beta-actin* gene of petunia as the reference gene [28]. Each experiment was repeated with three independent replications, and data are shown as the mean values ± SE (standard error). All primer pairs were designed by Primer Premier 5 software (Premier Biosoft Ltd., Palo Alto, CA, USA) (Table S3), the specificity of which was confirmed by sequencing of qRT-PCR products. The *beta-actin* gene was used as the internal control. 

### 4.7. Metabolite Extraction and Derivatization

For metabolite extraction, the petunia anthers (Stage 1, Stage 2 and Stage 3) stored at −80 °C were ground with a mortar and pestle in liquid nitrogen and transferred to 1.5-mL centrifuge tubes. Metabolites were extracted from the samples (ca. 50 mg) in 750 μL 100% methanol, and the samples were vortexed for 30 s. Then, 31.5 μL ribitol (0.2 mg mL^−1^) were added into each sample tube as an internal quantitative standard. The mixed samples were incubated for 15 min at 28 °C in an incubator shaker and then centrifuged for 10 min at 11,000× *g*. The supernatant (535 μL) of each sample was transferred into a 2-mL centrifuge tube, then 536 μL and 1071 μL ddH_2_O (double-distilled H_2_O) were added into each 2-mL tube. After being centrifuged (15 min, 2200× *g*), 400 μL supernatant of each sample were transferred to a new Eppendorf tube and immediately dried in a vacuum.

The residue was dissolved in 90 μL 20 mg·mL^−1^ methoxyamine pyridine solution and incubated for 120 min at 37 °C in an incubator shaker. Finally, the mixture was treated with 60 μL MSTFA reagent (containing 1% TMCS) and incubated for 30 min at 37 °C. Every experiment was repeated by six biological replicates.

### 4.8. GC-MS Analysis

A volume of 1 μL analyte for each sample was absorbed with a split ratio of 10:1 and injected into a DB-5 MS capillary column (30 m × 0.25 mm inner diameter, 0.25 μm film thickness; J&W Scientific, Folsom, CA, USA). The injection and transfer line temperatures were 280 and 270 °C. Helium was selected as the carrier gas, and the gas flow rate was 1 mL·min^−1^. The column initial temperature was retained at 70 °C for 5 min and finally increased to 300 °C at a heating rate of 5 °C·min^−1^ for 3 min. Following the same program of samples, the retention times of C10–C40 alkanes were obtained and used to generate retention indices (RIs) of sample peaks that have similarities of more than 700 with the spectral catalogs in NIST05 (National Institute of Standards). The peak area value of ribitol was taken as the internal standard to calculate the metabolite abundance. The SIMCA-P 11.5 software (Umetrics AB, Umea, Sweden) was used to examine the differences in the metabolite levels of petunia anthers of different development stages by partial least-squares discriminate analysis (PLS-DA). In order to confirm the significant differential metabolites at least between two different stages, the SPSS 13.0 software (SPSS Inc., Chicago, IL, USA) was used for variance analysis (*p* < 0.05). 

### 4.9. Statistical Analysis

Statistical analysis was performed using the SAS 8.1 software (SAS Institute, Cary, NC, USA) followed by Tukey’s multiple range tests (*p* < 0.01).

## 5. Conclusions

Based on the sequence data and mass chromatographic peaks obtained by GC-MS and RNA-Seq analysis at three typical development stages of petunia anthers, many critical unigenes and metabolites were identified, which showed that anther development is a complex process. The unigenes’ expression profiles and metabolite content changes through three petunia development stages were analyzed further. When combining the transcriptomic with GC-MS metabolomic results, the “starch and sucrose metabolism” pathway was indicated as the most important metabolic pathway in the petunia anther ripening processes. We found that the chlorophyll content, as well as the expression levels of the photosynthesis rate and chlorophyll biosynthetic genes all showed a decreasing trend in petunia anther ripening. The content of the two most important core different metabolites (fructose and glucose) all reached the peak in the microsporogenesis stage. These pieces of evidence suggested that the anther could be taken as a metabolic source, as well as a strong metabolic sink in the pollen mother cell stage, then it became only a weak sink in the following development stages, and the photosynthesis-related pathways could play important roles in the changes of the sink strength. This study offers new insights into the molecular and metabolic mechanisms in petunia anther development characteristics and could provide excellent platforms and study contents for future reproductive organ development research.

## Supplementary Materials

Supplementary materials can be found at http://www.mdpi.com/1422-0067/19/4/955/s1.
