# Supplementary material for: Transcriptomic and GC-MS Metabolomic Analyses Reveal the Sink Strength Changes during Petunia Anther Development
Source: Int J Mol Sci. 2018 Mar 23;19(4):955. doi: 10.3390/ijms19040955 (PMC5979359; doi:10.3390/ijms19040955)
Supplement: Supplementary file 1 [file ijms-19-00955-s001.zip › ijms-275446-suppl-final/Figures S1-S4.docx]

**
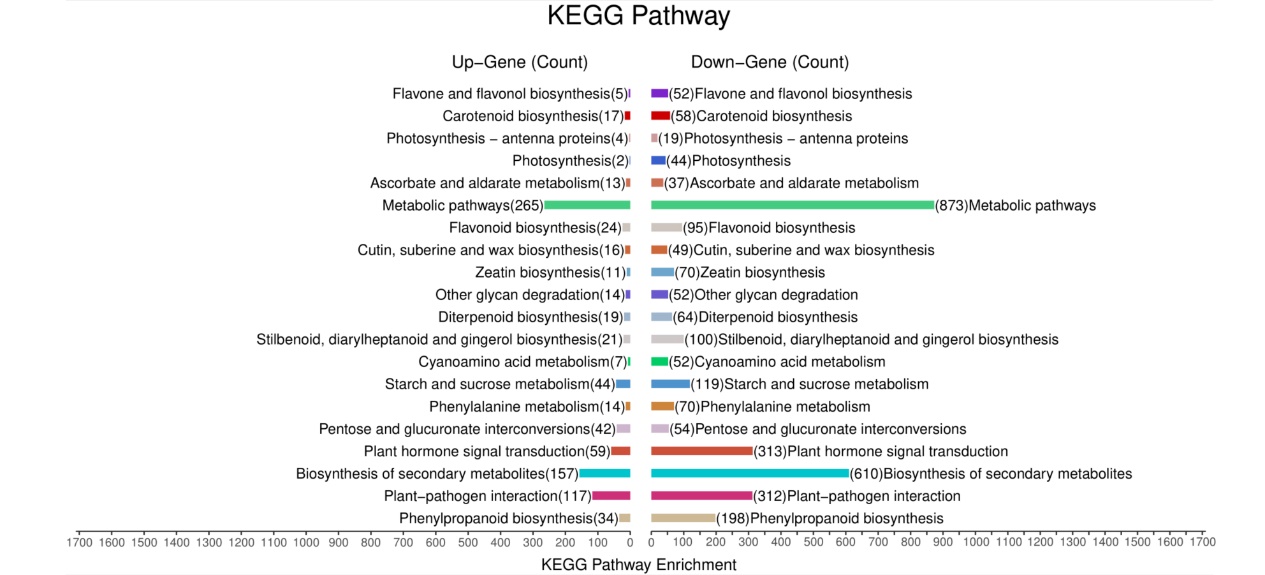

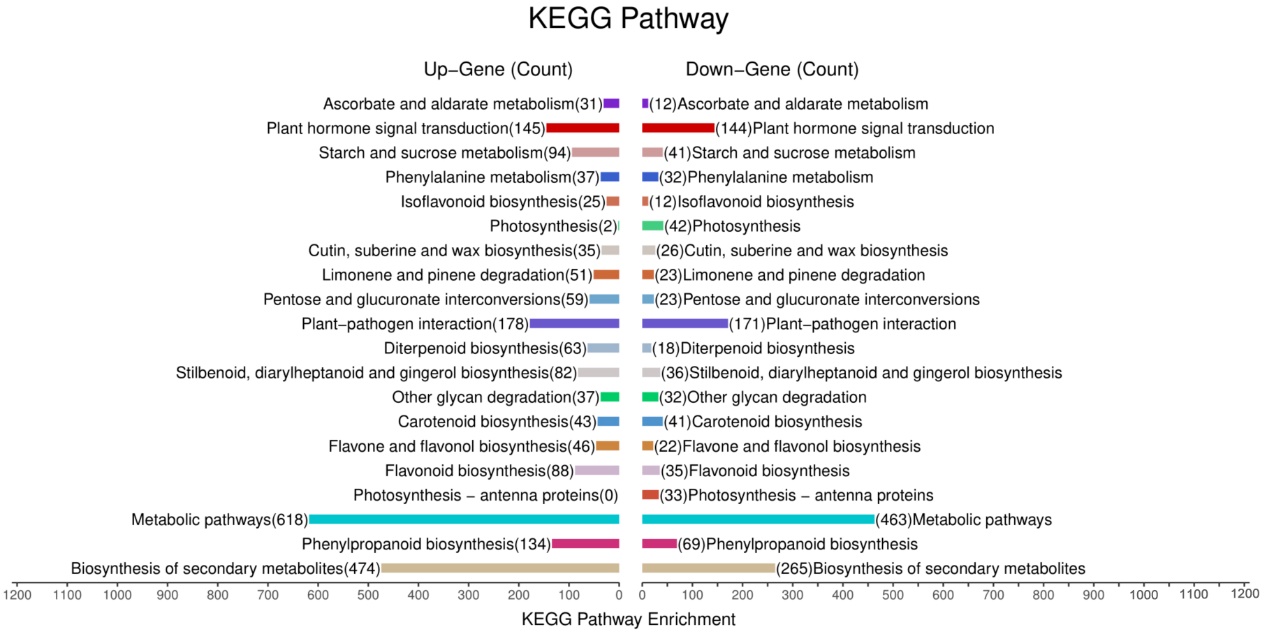
Figure S1.** The enriched KEGG pathways of up-regulated (left) or down-regulated (right) expressing genes of petunia anthers between stage1 and stage2.

**
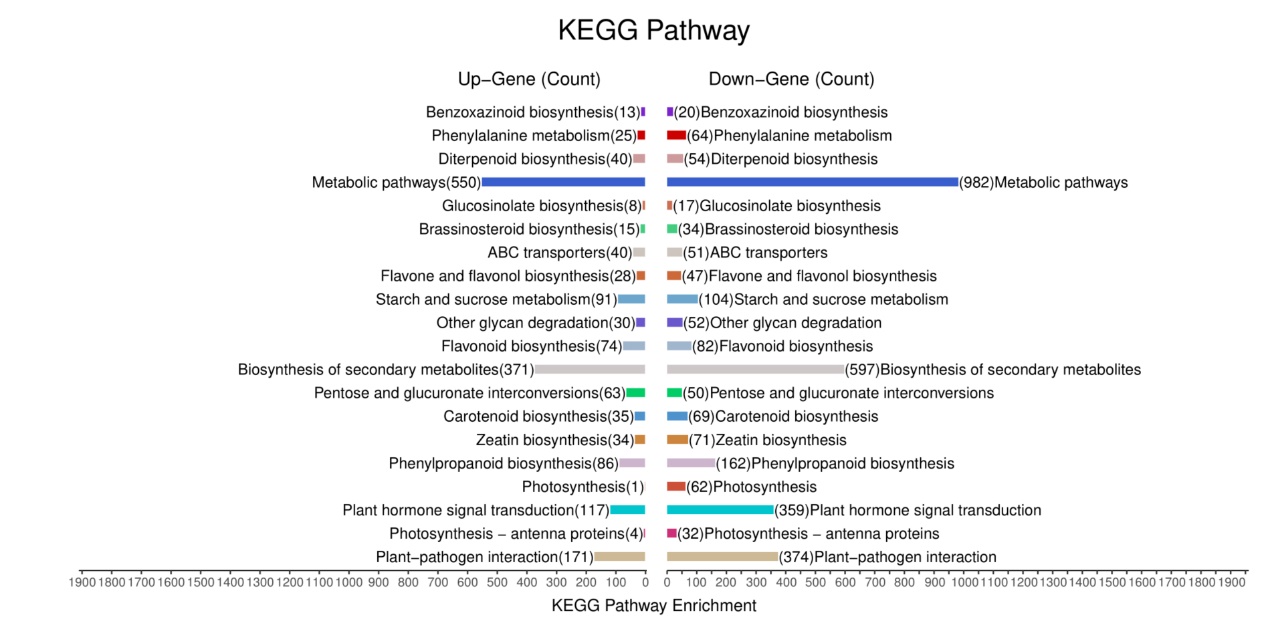
Figure S2.** The enriched KEGG pathways of up-regulated (left) or down-regulated (right) expressing genes of petunia anthers between stage2 and stage3.

**Figure S3.** The enriched KEGG pathways of up-regulated (left) or down-regulated (right) expressing genes of petunia anthers between stage1 and stage3.

**
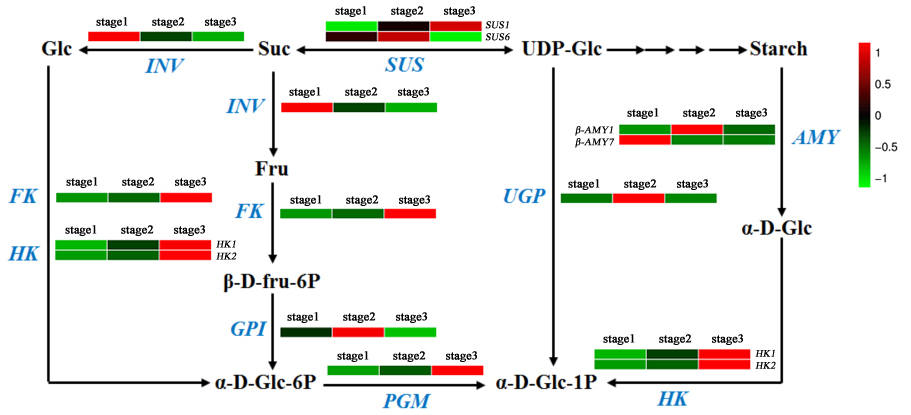
Figure S4.** The expression profiles of identified genes that participate in the putative starch and sucrose metabolism during petunia anther development. Suc, sucrose; Glc, glucose; *HK*, *HEXOKINASE*; *FK*, *FRUCTOKINASE*; *INV*, *INVERTASE*; β-D-fru, β-D-fructose; β-D-fru-6P, β-D-fructose-6-phosphate; *GPI*, *GLUCOSE-6-PHOSPHATE-ISOMERASE*; α-D-Glc-6P, α-D-glucose-6-phosphate; *PGM*, *PHOSPHOGLUCOMUTASE*; α-D-Glc-1P, α-D-glucose-1-phosphate; UDP-Glc, UDP-glucose; *SUS*, *SUCROSE SYNTHASE*; *UGP*, *UTP-GLUCOSE-1-PHOSPHATE URIDYLYLTRANSFERASE*; *AMY*, *β-AMYLASE*; α-D-Glc, α-D-glucose.
